# CMOS-Compatible Top-Down Fabrication of Periodic SiO_2_ Nanostructures using a Single Mask

**DOI:** 10.1186/s11671-015-1046-5

**Published:** 2015-08-26

**Authors:** Lingkuan Meng, Jianfeng Gao, Xiaobin He, Junjie Li, Yayi Wei, Jiang Yan

**Affiliations:** Institute of Microelectronics, Chinese Academy of Sciences, Beijing, 100029 People’s Republic of China

**Keywords:** CMOS-compatible top-down fabrication, Amorphous silicon mask, Periodic SiO_2_ nanostructures, Nanoline, nanotrench, and nanoholes, Nanotemplate, 81.16.Rf

## Abstract

We propose a CMOS-compatible top-down fabrication technique of highly-ordered and periodic SiO_2_ nanostructures using a single amorphous silicon (α-Si) mask layer. The α-Si mask pattern is precisely transferred into the underlying SiO_2_ substrate material with a high fidelity by a novel top-down fabrication. It is the first time for α-Si film used as an etch mask to fabricate SiO_2_ nanostructures including nanoline, nanotrench, and nanohole arrays. It is observed that the α-Si mask can significantly reduce the pattern edge roughness and achieve highly uniform and smooth sidewalls. This behavior may be attributed to the presence of high concentration of dangling bonds in α-Si mask surface. By controlling the process condition, it is possible to achieve a desired vertical etched profile with a controlled size. Our results demonstrate that SiO_2_ pattern as small as sub-20 nm may be achievable. The obtained SiO_2_ pattern can be further used as a nanotemplate to produce periodic or more complex silicon nanostructures. Moreover, this novel top-down approach is a potentially universal method that is fully compatible with the currently existing Si-based CMOS technologies. It offers a greater flexibility for the fabrication of various nanoscale devices in a simple and efficient way.

## Background

In recent years, periodic nanostructures are of great scientific interest and considerable technological importance and have been extensively investigated to meet the stringent requirement for many emerging applications, including biomedical sensors [1–3], phonics crystals [4, 5], photovoltaic devices [6, 7], surface plasmon resonance (SPR) sensors or surface-enhanced Raman scattering (SERS) [8–10], as well as nanoimprint template [11, 12]. There are numerous fabrication techniques and methods to produce periodic nanostructures of Si and SiO_2_ using top-down or bottom-up patterning strategies in the literatures. To fabricate successfully periodic nanostructures, it is easily understood that a most critical point is in achieving a precise pattern transfer with a high fidelity into the underlying substrate materials.

In general, it is particularly difficult to fabricate periodic SiO_2_ nanostructures with a good regularity and controllability in pattern size, roughness, and shape by a simple and efficient method, because it is difficult to find an appropriate mask and related excellent anisotropic etch process in nanometer scales. Furthermore, as chip architectures become increasingly complex, the use of hard mask to achieve a precise pattern transfer will be critically significant. Very often, multi-layer etch mask stacks composed of carbon-containing material, such as amorphous carbon or spin-on-carbon (SOC), and a few other material layers are widely applied in currently standard semiconductor nanofabrication [13, 14]. This kind of multi-layer stacks can improve the etch selectivity of photo resist to substrate materials during plasma patterning. However, the multi-layer mask plasma etch involves usually a complex process requiring expensive machinery and a very high process development cost. Any simplification of these processes offers a great advantage in both efficiency and cost, particularly for relatively small-scale production typical in research institutes.

In addition, due to the limitations of the conventional lithographic techniques, an excellent alternative such as the self-assembly of block copolymer has drawn a significant attention for the fabrication of periodic nanostructures, since it can access extremely dense and complex nanostructures with a low cost. Some periodic nanostructures of Si and SiO_2_ have been successfully fabricated with this technology in recent years [15–17]. Despite the numerous advantages and fruitful achievements of patterning nanostructures offered by the self-assembly, there are some challenges to restrict the patterning transfer of block copolymer patterns to various substrate materials. Among of them, etch resistance of block copolymer is inherently not strong enough as an etch mask to produce desirable nanostructures during pattern transfer using plasma etch. In this case, there have been some incompatible processes with currently mature semiconductor nanofabrication techniques used to fabricate various nanostructures. Most frequently, the metal film such as Cr or Au serving as an etch mask is very necessarily required to reduce the close dependence of dry etch process on the block copolymer, and also a lift-off process is generally required to define different patterns dependent on various specific requirements [17–20]. This makes the self-assembly difficult to be applied to currently standard semiconductor equipment, limiting mass-production and readily convenient integration into practical CMOS devices. Therefore, the technique is now not likely to be integrated into traditional semiconductor industry.

These pattern transfer routes presented above are generally complicated and incompatible with currently available semiconductor equipment and process. Thus, there is a critical requirement for a simple and efficient approach using readily available nanofabrication tool but with a capability for fabricating periodic nanostructures.

Here, we present a substantial improvement in etch mask technology to fabricate periodic SiO_2_ nanostructures by a novel, top-down, and CMOS-compatible fabrication. With the approach, a single amorphous silicon (α-Si) layer is used as an etch mask on the SiO_2_ substrate material to produce periodic nanostructures according to predefined nanopatterns using electron beam (e-beam) lithography. It is well known that α-Si has been widely used as a gate electrode material in CMOS or thin-film transistor for past many years. However, to the best of our knowledge, α-Si as a mask material used for the fabrication of nanostructures has not been investigated yet until now.

In previous studies [21, 22], a superior etch selectivity of SiO_2_ over α-Si material has been revealed in fluorocarbon-based plasma chemistry. It can be easily adjusted from low to even infinitely high values by optimizing process condition. It shows that α-Si material has a strong capacity to achieve a precise pattern transfer into the underlying substrate with a high fidelity, thus enables us to obtain an excellent process control. Actually, we have reported α-Si material used as a robust etch mask layer to fabricate successfully advanced 22-nm node planar device, 14-nm node finFET device, and some novel devices [23–25]. These devices fabricated show good device performances by the novel top-down approach.

Although, in this work, the fabrication of periodic SiO_2_ nanostructures is based on e-beam lithography using a top-down patterning strategy, it can easily extend to other lithography or bottom-up method such as self-assembly of block copolymer, because a superior etch selectivity of SiO_2_ over α-Si is a key factor that enables this approach. More significantly, the fabrication process is simple and fully CMOS-compatible with existing silicon integrated circuit technology, and therefore, it can be easily incorporated and integrated into standard semiconductor nanofabrication and is easily scalable in a simple and efficient way.

## Methods

**Fig. 1:**
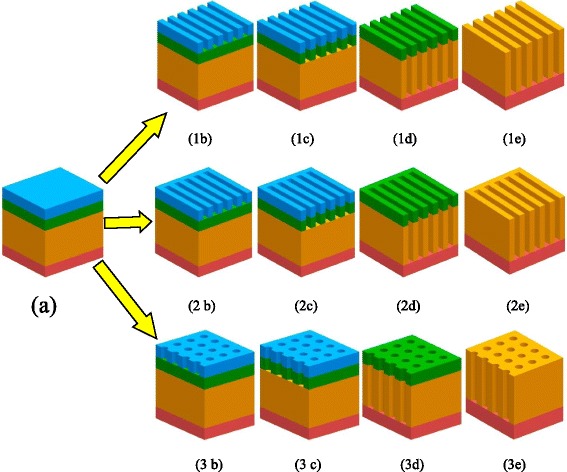
Schematic of the process for fabricating periodic SiO_2_ nanostructures including nanoline, nanotrench, and nanohole arrays. *a* SiO_2_ nanostructures sample preparation, *1b-1e* nanoline arrays fabrication process: *1b* e-beam lithography; *1c* α-Si mask is opened using the resist mask by RIE; *1d* SiO_2_ nanoline arrays are produced by RIE; *1e* α-Si mask is selectively removed using wet etch in TMAH solution. *2b-3e* Nanotrench and nanohole arrays fabrication process using the same approach as *1b-1e*

All experiments were conducted on 200-mm single crystal silicon substrates (p-type, (100), 1–10 Ω cm). Figure 1 shows a schematic drawing of the novel fabrication process for making periodic SiO_2_ nanostructures including nanoline, nanotrench, and nanohole. A SiO_2_ layer, which was deposited on bulk silicon substrate using plasma-enhanced chemical vapor deposition (PECVD) followed by a 50-nm-thick α-Si thermally grown by rapid thermal processing (RTP). The e-beam resist was then spin-coated on the underlying α-Si mask by Kingsemi automatic track (Fig. 1a). To obtain a highly dense nanoline and nanotrench arrays with 40-nm line width and 40-nm spacing, the electron beam exposure was performed on a Gaussian (spot) beam system, NBL NB5, at an acceleration voltage of 80 kV and beam current of 2 nA with small beam spot size that were used to write the AR-N-7520 resist (negative and non-chemically amplified resist). The e-beam dose was 500 μC/cm^2^ for the exposure of line and spacing pattern. After e-beam writing, post-exposure baking (PEB) of 75 °C was applied for 120 s. The wafers were developed for 60 s in 2.38 % TMAH (tetramethylammonium hydroxide) developer and then rinsed with DI water. Post-development baking (PDB) of 130 °C for 120 s for further drying and hardening of e-beam resist was applied.

Similarly, to produce highly ordered nanohole arrays with a diameter of 45 nm, the electron beam exposure was performed at an acceleration voltage of 80 kV and beam current of 3 nA to write the ZEP520 e-beam resist (positive and non-chemically amplified resist). The e-beam dose was 500 μC/cm^2^ for the exposure of hole pattern. After e-beam writing, the wafers were developed for 60 s in ZED-N50 developer and then rinsed with MIBK (methyl isobutyl ketone).

Then, the resist patterns were transferred into the underlying α-Si film by commercial inductively coupled plasma (ICP) etch tool Lam TCP 9400DFM using Cl_2_/HBr/O_2_ plasma chemistry. The resulting etched α-Si requires selectively to stop on the underlying SiO_2_ substrate by a sufficiently high etch selectivity over the resist using an appropriate reactive ion etch (RIE) process condition (Fig. 1c). Very clearly, it is a critical step that plays a significant role in affecting the resulting etched width of SiO_2_ nanostructures. After the resist was removed, SiO_2_ features can be achieved by Lam Exelan HPT etch tool using C_4_F_6_/CO/Ar/O_2_ fluorocarbon-based plasma chemistry (Fig. 1d). According to specific requirement, remaining α-Si mask can be selectively removed without a damage on the underlying substrate layer by wet etch in TMAH solution (Fig. 1e).

The top and cross-sectional characterizations of fabricated nanostructures were carried out using a Hitachi scanning electron microscopy SEM4800 and SEM5500, respectively.

## Results and Discussion

**Fig. 2:**
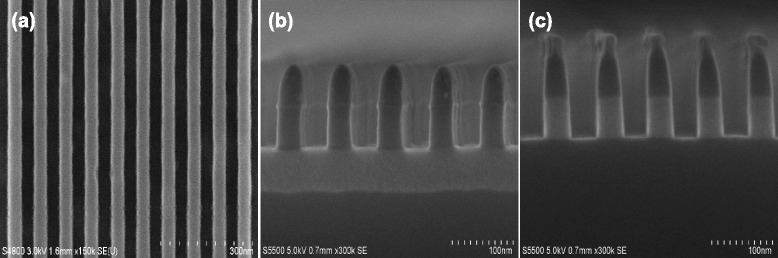
SEM images of the fabrication of highly ordered and periodic SiO_2_ nanoline arrays with 40 nm line width and 40-nm spacing. **a** The resist nanoline arrays are patterned by e-beam lithography, and the bright area is the line. **b** Nanoline patterns are precisely transferred into the underlying α-Si mask. **c** SiO_2_ nanoline arrays are directly formed only by a single α-Si mask without complex patterning structures

Figure 2 shows a top-down and cross-sectional SEM images of SiO_2_ nanoline arrays on the silicon substrate only by a single α-Si material serving as an etch mask. It is well known that α-Si material demonstrates a fairly high etch selectivity over the resist film in halogen-based plasma chemistry, such resulting in a good pattern transfer fidgety. As shown in Fig. 2b, it can be seen that highly ordered and 50-nm thick α-Si mask patterns have been achieved successfully with smooth sidewalls without any deformation, twisting, or collapse. In addition, it is noted that, the α-Si mask indicates an almost vertical etched profile with only slightly tapered sidewall angle. Then, the resulting etched SiO_2_ nanoline arrays with 40 nm width, a period of 80 nm, and a height of 50 nm have been smoothly fabricated using appropriate fluorocarbon-based plasma chemistry. Smooth and nearly vertical etched sidewalls demonstrate an almost perfect pattern transfer from the α-Si mask. After patterning SiO_2_, the top α-Si mask layer is not damaged or attacked at all in vertical direction except a slightly lateral loss at the top of the layer. It implies a relatively high etch selectivity between them capable of fabricating higher aspect ratio nanoline arrays by selecting an appropriate mask thickness. In addition, it can be expected that SiO_2_ nanoline arrays with a smaller width and period may be achieved by the approach proposed above combined with a more aggressive lithography technology such as self-assembly of block copolymer, since SiO_2_ patterning capacity is inherently determined by etch selectivity between SiO_2_ and α-Si mask layer. In present, the fabrication of SiO_2_ nanoline arrays with more aggressive etched characteristic is being investigated and will be reported in a future publication.

Owing to the high etch resistance, the SiO_2_ arrays fabricated can be directly used as an etch mask to transfer periodic nanoline arrays to the substrate materials underneath by using conventional reactive ion etch capable of producing specifically required nanostructures. For example, it can be applied to fabricate silicon nanoline arrays with an outstanding merit, since a high etch selectivity of silicon over SiO_2_ can be easily obtained in halogen-based plasma chemistry. Furthermore, the remaining top α-Si mask after SiO_2_ patterning can be easily removed during silicon etch process, since there is a similar etch property between α-Si mask and silicon. This will significantly avoid the use of an extra process and further enhance the process window.

**Fig. 3:**
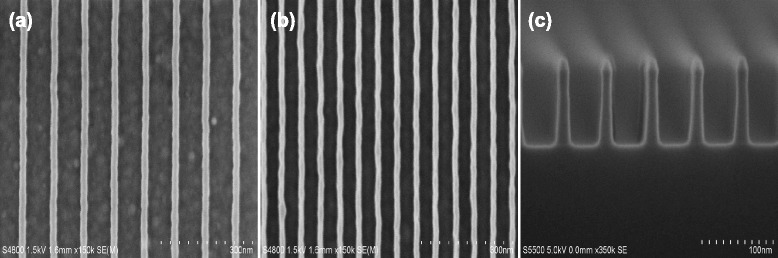
SEM images of the fabrication of periodic silicon nanoline arrays with 20 nm line width and 40-nm spacing. **a** Arrays of the resist nanoline with a width of 20 nm are patterned by e-beam lithography, and the bright area was the line. **b** Arrays of α-Si mask nanoline are fabricated by a precise pattern transfer in ICP etcher by Cl_2_/HBr/O_2_ plasma chemistry. **c** The silicon nanoline arrays are successfully fabricated by a high fidelity pattern transfer from α-Si mask and SiO_2_ nanotemplate, demonstrating a nearly vertical etched profile as well as the smooth sidewalls

Figure 3 demonstrates a typical fabrication applied in photonic devices or nanofluidic devices by silicon arrays. Due to resolution limit of e-beam resist, for the resist patterns with the line width of 20 nm, there are considerably numbers of burrs that can be found in the line edge. Here, we first fabricate the SiO_2_ nanotemplate using the same approach proposed above, and then it is precisely transferred into the underlying silicon substrate to produce periodic silicon nanoline arrays in halogen-based plasma chemistry by a mixture of Cl_2_ and HBr. As shown in Fig. 3c, it is observed that highly ordered and periodic silicon arrays with smooth sidewalls have been successfully achieved, having a line width of 20 nm, a period of 60 nm, as well as a fairly high aspect ratio structure of near 5:1 with almost vertical etched profiles. Higher aspect ratio silicon arrays can be easily achieved by increasing thickness of SiO_2_ film without the requirement of adding an extra process. It indicates that the ultimate limit of etched feature size is determined by the patterning ability of lithography technology rather than the approach itself proposed in this work.

In addition, it is clearly observed that α-Si nanoline sidewalls are much smoother than those of the resist patterns. It strongly reveals that α-Si may play a significant role in reducing line edge roughness. Line edge roughness is an important factor contributing to the device degradation in various nanoscale devices. Here, the improvement of line edge roughness may be closely related to the dangling bonds at the surface of α-Si, and an in-depth investigation is still being done. It is well established that dangling bonds are created. It is well known that the silicon atom possesses four valence electrons and therefore requires four bonds to fully saturate the valence shell. In the crystalline structure silicon, each silicon atom establishes bonds to its four neighboring atoms, leaving no unsaturated bond behind. However, in the case of α-Si, the amorphous crystalline structure silicon displays a high concentration of dangling bonds. Some silicon atoms in the structure and at the surface are missing and remain unbonded, leading to a lack of a long-range order. These dangling bonds can be easily passivated by incorporating atomic hydrogen to saturate them, thereby improving the quality of the material [26, 27]. Here, atomic hydrogen provided by HBr gas is a main source for α-Si mask patterning, and therefore, the passivation of dangling bonds at the surface of α-Si is easily achieved. Furthermore, this will make the sidewall of α-Si mask be more resistive for etch process and then make line edge roughness be significantly reduced to achieve smooth sidewalls.

With the novel technique, α-Si have demonstrated a great potential for the smoothness of sidewalls for the fabrication of nanostructures with significantly reduced line edge roughness. Compared with other smoothing techniques previously reported, our approach is efficient and easy to implement. In fact, the smooth sidewall with vertical etched profiles will be critically beneficial to reduce optical scattering losses for photonic crystals strongly dependent on the sidewall surface [28, 29]. By increasing the mask thickness, a much larger aspect ratio and etched depth can be expected. Furthermore, the extremely smooth surfaces of these nanostructures lead to the realization of excellent electrical performance.

**Fig. 4:**
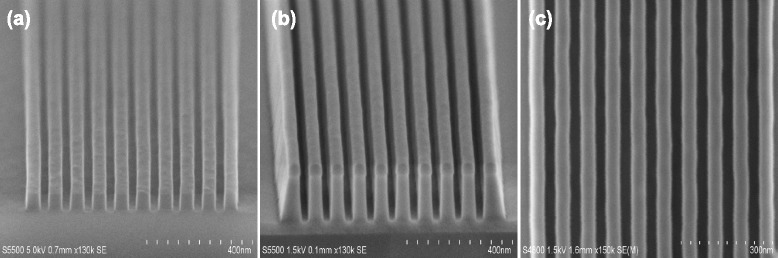
SEM images of the fabrication of periodic silicon nanotrench arrays with 40 nm line width and 40-nm spacing. **a** Arrays of α-Si mask nanotrench are patterned by a precise pattern transfer in ICP etcher by Cl_2_/HBr/O_2_ plasma chemistry. **b** SiO_2_ nanotrench arrays fabricated show a highly uniform and vertical etched profile. **c** *Top view* of (b) showing a highly smooth sidewalls

Figure 4 shows cross-sectional SEM images of a highly ordered and periodic SiO_2_ nanotrench arrays fabricated using a single α-Si mask with the same e-beam lithography condition as described in Fig. 2. As presented above, to create SiO_2_ nanotrench arrays, α-Si mask is firstly patterned by a mixture gas of Cl_2_/HBr/O_2_ in an ICP chamber, as shown in Fig. 4a. Note that here, it should be pointed out that the remaining resist film after α-Si mask patterning is very necessarily removed in order to avoid a not good influence on the fabrication of SiO_2_ nanotrench arrays. Otherwise, in this case, it will be very difficult to obtain smooth and desirable SiO_2_ features, and a severe patterning distortion is easily produced. The resist removal can be achieved by an O_2_ plasma ashing in combination with a wet cleaning process composed of a dip in dilute hydrofluoric acid (DHF) followed by sulfuric peroxide mixtures (SPM). Then, the periodic α-Si patterns formed by the resist are transferred into the underlying SiO_2_ film in the LAM Exelan Hpt etcher. Pattern transfer with a high fidelity into the underlying SiO_2_ film to create a high aspect ratio structure is always a challenge in nanometer scales. Here, an optimized process condition is developed to achieve a good pattern transfer using a fluorocarbon-based plasma chemistry including C_4_F_6_/CO/O_2_/Ar mixture gases. As shown in Fig. 4b, c, it is evident that the resulting nanotrench arrays retain highly ordered and periodic nanostructure with an opening width of around 35 nm. The cross-sectional view of nanotrench arrays demonstrates that all etched SiO_2_ trenches are highly uniform and smooth, showing an excellent uniformity and high reproducibility by simply using the α-Si mask. The etched depth of the SiO_2_ nanotrench can be easily controlled by processing time. Figure 4b, c shows that SiO_2_ nanotrench arrays fabricated have a width of 35 nm with a nearly vertical etched profile as well as fairly smooth sidewalls.

**Fig. 5:**
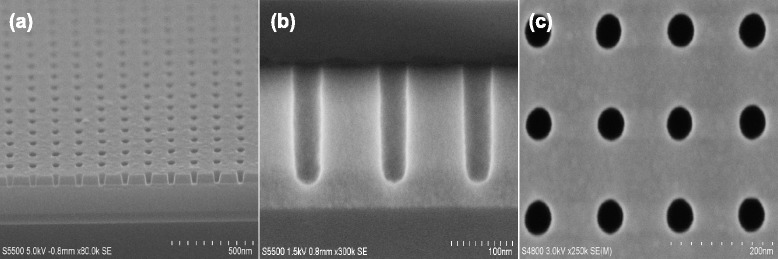
Cross-sectional and top-down SiO_2_ nanohole SEM views. **a** Arrays of α-Si mask nanohole are patterned by Cl_2_/HBr/O_2_ plasma chemistry. **b** SiO_2_ nanotrench arrays fabricated show an almost vertical etched profile with smooth sidewalls, and here, the α-Si mask has been removed selectively. **c** *Top view* of (b) showing a highly uniform etch performance

Similarly, besides providing a much simpler approach than conventional patterning strategy for generating nanotrench arrays, the straightforward and CMOS-compatible approach enables a facile creation of SiO_2_ nanohole arrays that will be not easily available by traditional single mask etch technology. Figure 5 shows that the arrays of high-quality and periodic nanohole with around 45 nm diameter and 140-nm period have been fabricated successfully using the same process condition as described in Fig. 4. It implies that the simple process is easily extended to different fabrications of SiO_2_ nanostructures. In Fig. 5b, the SiO_2_ nanohole arrays clearly indicate that all etched holes are almost perfectly straight and highly uniform, demonstrating a very good etch controllability.

In this work, it can be easily observed that α-Si is only used as an etch mask served as a sacrificial layer, and it will not be left on final structure. As a consequence, it will not pose a not good effect on electrical device performance. On the contrary, the novel nanofabrication technique shows some advantages than some previously reported ones.

## Conclusions

A novel and simple approach for fabricating periodic SiO_2_ nanostructures with highly smooth sidewalls has been demonstrated using a single α-Si mask layer. It is a fully CMOS-compatible new strategy to transfer the resist pattern into the underlying mask by top-down approach. These patterns are then used as an etch mask to fabricate SiO_2_ nanostructures including nanoline, nanotrench, and nanohole arrays. It is observed that α-Si mask plays a significant role in reducing line edge roughness and contributes to successful realization of smooth sidewalls without using any extra process treatment step. Using the proposed technology, we have successfully fabricated SiO_2_ and silicon nanoline arrays about 40 and 20 nm in width, respectively, having nearly vertical etched profiles with smooth sidewalls. In addition, highly ordered and periodic nanotrench and nanohole arrays with 35 and 45 nm in width are fabricated, showing an excellent result. These results show that the SiO_2_ patterns as small as sub-20 nm may be achievable using the simple top-down fabrication. The proposed method not only simplifies the fabrication process but also efficiently produces periodic nanostructures. The novel technology is fully compatible with the current Si-based CMOS technologies and provides a general approach for fabricating simple and complex nanostructures.
